# EAST MEETS WEST: COLLABORATING INTERNATIONALLY TO MITIGATE SOCIAL ISOLATION

**DOI:** 10.1093/geroni/igae098.1888

**Published:** 2024-12-31

**Authors:** Megumi Inoue, Takashi Amano, Jim Lubben

**Affiliations:** Chair; Co-Chair; Discussant

## Abstract

Combatting social isolation in later life is a pressing global issue. This symposium assesses social isolation among older adults from various angles. Four presenters from North America and Japan examine the perception, disparities, and impact of social isolation within specific cultural contexts. The knowledge from diverse cultural backgrounds and policy frameworks of the different countries provides an opportunity to explore the intricate nuances and shared experiences of social isolation among older populations. Dr. Watanabe will introduce the status of social isolation among Japanese older adults who reside in a marginal community, where more than 50% of the population is 65 years or older. Dr. Murayama will describe the findings on different forms of social isolation and their impact on health outcomes, utilizing extensive cohort data from older adults living in the Tokyo metropolitan area. Dr. Taylor will examine disparities in loneliness among older adults in the U.S., with a particular focus on the mediation effects of socioeconomic factors on racial and ethnic differences in loneliness. Dr. Mair will discuss changing demographics of older adults and shifting cultural values from a global perspective, with implications for rethinking aging alone as a risk factor and traditional notions of successful aging in relation to limited family ties. These four presentations will provide a broad overview of social isolation in three countries and its implications for quality of life in later life. The symposium will help identify culturally-sensitive strategies for promoting the well-being of older adults in global communities.

